# Biochemical and genomic evidence for converging metabolic routes of metformin and biguanide breakdown in environmental Pseudomonads

**DOI:** 10.1016/j.jbc.2024.107935

**Published:** 2024-10-28

**Authors:** Katie B. Wissbroecker, Anthony J. Zmuda, Harsheeth Karumanchi, Thomas D. Niehaus

**Affiliations:** The Department of Plant and Microbial Biology, University of Minnesota, Minneapolis, Minnesota, USA

**Keywords:** metformin, drug metabolism, *Pseudomonas*, hydrolase, microbiome, bacterial metabolism

## Abstract

Metformin is commonly used to lower blood glucose levels and is one of the most widely used pharmaceuticals worldwide. Typical doses are high (0.5–2.0 g day^−1^) and the majority travels through the digestive system unabsorbed and enters the wastewater system. Metformin is not removed by standard wastewater treatments and eventually enters freshwater systems, where it can form *N*-chloro-derivatives that are toxic to fish and human cells. Thus, metformin is one of the most prevalent anthropogenic pollutants worldwide and there has been considerable interest in finding ways to remove it. We recently isolated Pseudomonads capable of growing on metformin as the sole nitrogen source. We identified candidate genes involved in metformin breakdown through genomic analyses informed by feeding studies. One candidate, a pair of genes that are located on ∼80kb extra-genomic plasmids, was shown to encode a heteromeric Ni-dependent hydrolase that converts metformin to guanylurea and dimethylamine. Metforminase activity of these gene products is now well established as our results confirm three recently published independent studies. Our isolated Pseudomonads also grow on biguanide, suggesting the existence of an additional breakdown enzyme. Another candidate gene located on the ∼80kb plasmids was shown to encode an aminohydrolase that converts biguanide to guanylurea. Biguanide may arise through successive *N*-demethylations of metformin or come from other sources. Our results suggest that the recent evolution of metforminase and biguanide hydrolase enzymes allow Pseudomonads to convert either metformin or biguanide to guanylurea, which can be assimilated by existing pathways.

The biguanide-derivative metformin is a common therapeutic for treating type 2 diabetes and is one of the most widely used drugs in the world with over 250 million daily prescriptions; this is expected to increase to an estimated 783 million prescriptions by 2045 according to the IDF Diabetes Atlas 10th Edition. Metformin is generally effective at lowering blood glucose levels when administered orally by causing the inhibition of gluconeogenesis in the liver (1, 2, 3). Despite decades of investigations, the mode of action of metformin is still not fully understood (4). However, there is strong, mounting evidence for the involvement of gut microbes to exert a blood glucose lowering effect (4, 5, 6, 7, 8). It was recently shown that metformin is a competitive inhibitor of the gut microbial enzyme agmatinase and that impaired agmatine catabolism in the gut could play a role in metformin’s therapeutic effects (9). Metformin also reportedly has antiaging (10), antiobesity (11, 12, 13), and antitumor properties (14), suggesting that metformin usage could expand beyond the treatment of type 2 diabetes (15).

Daily doses of metformin are high (0.5–2.5 g day^−1^) and the vast majority pass through the body unchanged and are removed in urine or feces where it enters the wastewater steam (16, 17, 18). Wastewater treatment plants are ineffective at removing metformin because it is not absorbed by activated carbon and chlorination forms *N*-chloro-metformin derivatives that are toxic to human cells and fish (19, 20, 21). Because of the high dosage and its recalcitrance, more than 100 million kg of metformin enter the environment each year, making metformin one of the most pervasive anthropogenic pollutants today (22, 23).

There is strong ecological and medical interest in understanding metformin metabolism because the therapeutic effects of metformin are linked to gut microbiota (4, 5, 6, 7, 8) and because of its pervasiveness in the environment (22, 23). Metformin breakdown mediated by microorganisms has been detected in activated sludge of wastewater treatment plants, with guanylurea being the most widely detected breakdown product (24, 25, 26, 27, 28). Guanylurea has also been detected in rats on metformin regimens, possibly due to metformin breakdown by gut microbiota (8, 29). Isolation from activated sludge of a single species that can fully metabolize guanylurea led to the identification of a hydrolase (GuuH) that converts guanylurea to guanidine (Fig. 1) (30). Guanidine can be fully metabolized to carbon dioxide and ammonia by the guanidine carboxylase pathway (Fig. 1) (31, 32).

**Figure 1:**
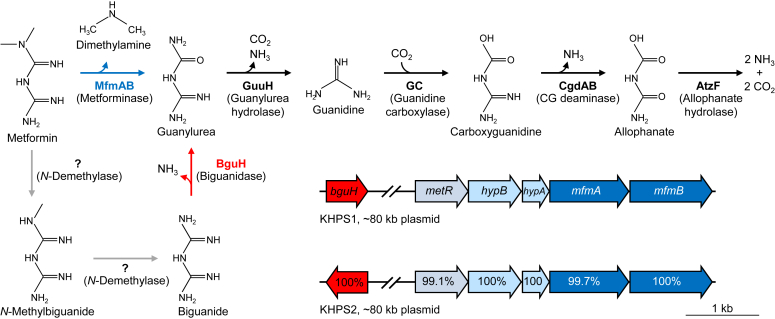
**Metformin breakdown pathways in KHPS1 and KHPS2 *Pseudomonas* isolates.** The genomes of the metformin-utilizing isolate *Pseudomonas* sp. KHPS1 and *Pseudomonas hydrolytica* strain KHPS2 encode previously characterized enzymes involved in the complete breakdown of guanylurea (*black arrows*). Genomic analyses identified candidate genes involved in metformin breakdown that are encoded on ∼80 kb extrachromosomal plasmids. Metforminase genes (*mfmAB*, colored *blue*) are in an operon with genes encoding predicted Nickel incorporation proteins (*hypAB*) and a predicted MetR family transcriptional regulator. Also encoded on these plasmids is a biguanide hydrolase gene (*bguH*, colored *red*). The amino acid percent identity between orthologues is indicated. Hypothetical reactions are shown with *gray arrows*.

Recently, bacterial strains that grow on metformin as the sole nitrogen or carbon source have been isolated by our group (33), as well as independent research groups in North America (34), Europe (35), and China (36). Genomic analyses of these strains led to the identification of a heteromeric metforminase that converts metformin to guanylurea and dimethylamine (37, 38, 39). Discovery of this enzyme completes a metformin assimilation pathway (Fig. 1). This heteromeric metforminase is conserved in all reported metformin-utilizing bacterial isolates, suggesting it is quickly becoming the primary means to initiate metformin breakdown.

Here we report on the investigations into metformin breakdown in our *Pseudomonas* isolates. We confirm that a heteromeric metforminase converts metformin to guanylurea in our strains. We also observed that our isolates could grow on biguanide and we identified a novel aminohydrolase that converts biguanide to guanylurea, demonstrating the use of converging metabolic routes to degrade metformin or other biguanide derivatives.

## Results

### Genomic analysis of *Pseudomonas* isolates

The partial characterization of metformin breakdown pathways and ecological studies suggest that metformin breakdown proceeds through a guanylurea intermediate (24, 25, 26, 27, 28, 30). The chromosomal genomes of both *Pseudomonas* sp. KHPS1 and *Pseudomonas hydrolytica* strain KHPS2 encode enzymes capable of the complete assimilation of guanylurea into carbon dioxide and ammonia (Fig. 1) (30, 31, 32, 34). In addition to the chromosomal genome, both isolates contained ∼80 kb plasmids (KHPS1, 79.8 kb; KHPS2, 83.8 kb), each with about 90 protein encoding genes (33). As recently evolved traits that provide a fitness advantage to microbial communities are commonly plasmid-encoded (40), we searched plasmid sequences for genes involved in the conversion of metformin to guanylurea. Two candidate metforminases were identified. The first is encoded by a pair of tandem genes (denoted *mfmAB*) that encode ureohydrolase family proteins, which typically catalyze hydrolysis reactions resulting in the release of urea (41). A similar reaction could conceivably convert metformin to guanylurea, except that dimethylamine would be released instead of urea. *mfmAB* are located in an operon with genes predicted to encode nickel chaperone proteins, *hypAB* (42), and a MetR family transcriptional regulator (43) (Fig. 1). Close homologs of *mfmAB* are found on either plasmids or chromosomal genomes of recently identified *Pseudomonas* and *Aminobacter* isolates that can grow on metformin as a nitrogen and/or carbon source (34, 35, 36). Thus, genomic context and reaction mechanisms of ureohydrolase family proteins both support *mfmAB* as a good metforminase candidate.

The other candidate is a predicted nucleoside aminohydrolase (denoted *bguH*) also encoded on the plasmids of both isolates. An aminohydrolase could also conceivably be involved in metformin breakdown, although not *via* the direct conversion of metformin to guanylurea. Close homologs are not found in other recently identified species that can metabolize metformin, except for another strain isolated from a similar location in Minnesota around the same time our isolates were identified (34). Nonetheless, we considered *bguH* a viable metformin breakdown candidate.

### Growth of *Pseudomonas* isolates on biguanide derivatives

**Figure 2:**
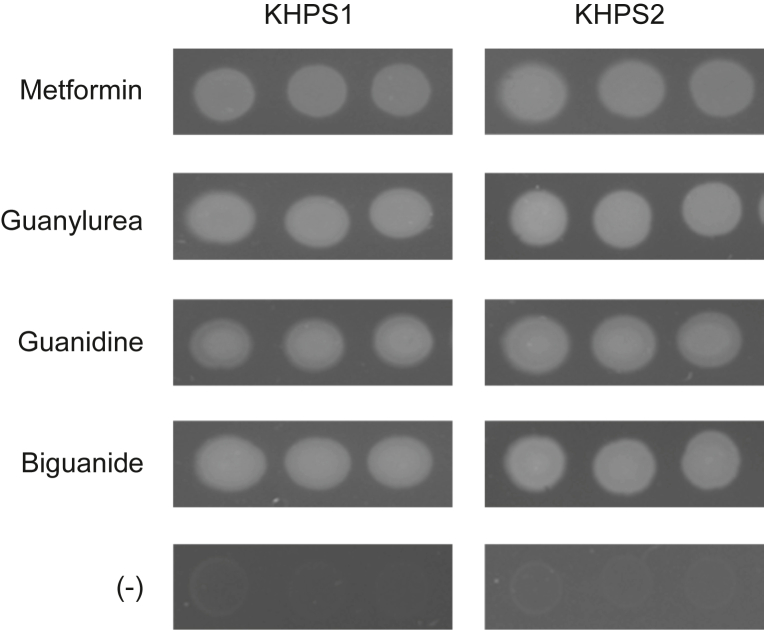
***Pseudomonas* isolates use metformin and its breakdown products as nitrogen sources.***Pseudomonas* sp. KHPS1 and *Pseudomonas hydrolytica* strain KHPS2 were grown overnight in LB medium, washed thrice with citrate-acetate minimal medium lacking nitrogen, and diluted to an optical density of 1.0, 0.2, and 0.04. 5 μl of each dilution was spotted on minimal medium plates containing 1 mM of metformin, guanylurea, guanidine, or biguanide as the only nitrogen source, or minimal medium lacking nitrogen (−). Plates were incubated for 24 h at 37 °C prior to imaging.

*Pseudomonas* sp. KHPS1 and *P. hydrolytica* strain KHPS2 were isolated from primary sludge from the St Paul water treatment plant by screening for the ability to grow on metformin as the sole nitrogen source (33). Genomic analysis indicated that both isolates possess the genes for complete guanylurea assimilation, which we confirmed with feeding studies (Fig. 2). In addition to metformin, both isolates were able to grow on the major predicted metformin breakdown product guanylurea, as well as its predicted breakdown product guanidine (Fig. 2). Both isolates grew well on biguanide (Fig. 2), which is a possible metformin breakdown product that could arise from successive *N*-demethylations of metformin, with *N*-methylbiguanide as the intermediate (Fig. 1). Both isolates could use *N*-methylbiguanide as a nitrogen source, although not as effectively as metformin (Fig. S1). Growth was saturated after 24 h growth on minimal medium plates supplemented with metformin, biguanide, or their breakdown products (Fig. 2). However, growth was not yet saturated after 48 h of growth on *N*-methylbiguanide (Fig. S1). These growth assays suggest that both of our isolates are capable of breaking down metformin *via* a predicted pathway involving the transformation of metformin to guanylurea. The use of biguanide, and to a lesser extent *N*-methylbiguanide, suggests that an additional pathway may also be present.

### Screening metforminase candidate genes

**Figure 3:**
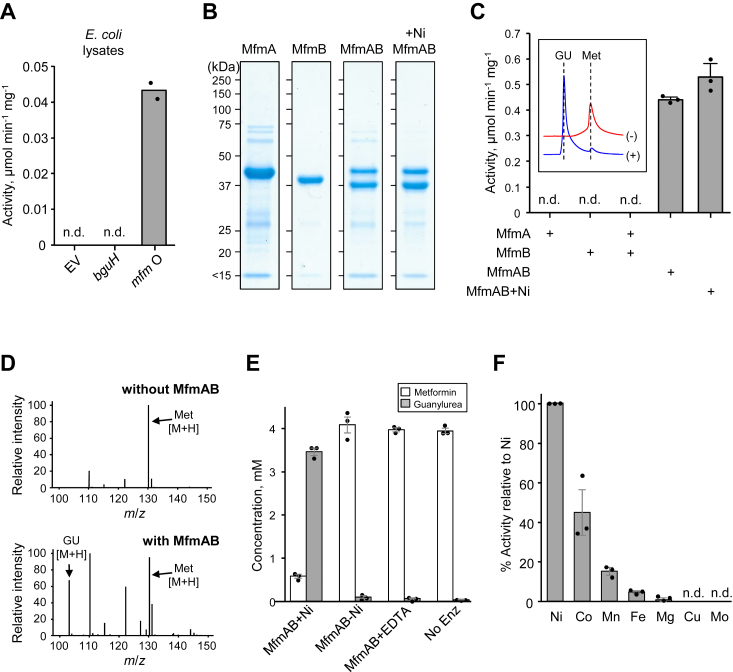
**Metforminase activity of *mfmAB* coexpression product.***A*, metforminase activity in soluble protein lysates of *E. coli* harboring pUC19 with the indicated gene(s). Assays (100 μl total volume) contained 50 mM Tris-HCl, pH 8.0, 4 mM metformin, 0.5 mM NiCl_2_ and other divalent cations (see methods), 95 μg total lysate protein, and were incubated at 37 °C for 1.5 h prior to HPLC analysis. Data represents the average of 2 biological replicates. *B*, purity of enriched MetA and MetB expressed separately or co-expressed in *E. coli* without or with addition of 0.5 mM NiCl_2_ to cultures at induction (+Ni). *C*, Metforminase activity of purified MetA, MetB, and MetAB proteins expressed without or with the addition of nickel at induction (+Ni). Assays (100 μl total volume) contained 125 mM sodium phosphate, pH 8.0, 4 mM metformin, 2 mM NiCl_2_, 5 μg protein, and were incubated at 37 °C for 2 h prior to HPLC analysis. Data represents the mean and S.E.M. of 3 independent replicates. The inset shows representative HPLC traces at 220 nm of assays without (-, *red* trace) and with the addition of MfmAB (+, *blue* trace). *D*, LC-MS analysis of MfmAB reaction products shows the appearance of guanylurea. Assays (100 μl total volume) contained 100 mM ammonium bicarbonate, pH 8.0, 2.5 mM metformin, 0.5 mM NiCl_2_, without or with 5.0 μg MfmAB, and were incubated at 37 °C for 2 h prior to LC-MS analysis. *E*, results of LC-MS analysis of assays without or with nickel or with EDTA shows metal dependence of MfmAB. Assays were as described in (*D*) except for the presence or absence of NiCl_2_ and 5 mM EDTA. Data represents the mean and S.E.M. of three independent replicates. *F*, activity of MfmAB incubated with various metals. Assays were as described in (*C*) except that NiCl_2_ was replaced by the indicated metal. Data represents the mean and S.E.M. of three independent replicates.

As an initial screen of our two metforminase candidates, we expressed each in *Escherichia coli* and assayed cell lysates for metforminase activity. Since the candidate *mfmAB* occurred in a conserved operon with predicted nickel insertion proteins, we cloned the entire *hypBAmfmAB* operon into pUC19. The other candidate *bguH* was also cloned into pUC19. *E. coli* MG1655 harboring each construct or empty vector were grown in LB medium. Whole cell lysates were prepared and used in an HPLC-based enzyme assay in which metformin and the expected reaction product guanylurea are resolved chromatographically and quantified by UV absorbance. Reaction mixtures contained 50 mM Tris-HCl, pH 8.0, 4 mM metformin, 0.5 mM NiCl_2_ and other divalent cations (see methods), 95 μg total lysate protein, and were incubated at 37 °C for 1.5 h prior to analysis. Metforminase activity was observed in lysates of *E. coli* harboring *hypBAmfmAB*-pUC19 (Fig. 3*A*). We detected no metforminase activity in lysates from *E. coli* harboring empty vector or *bguH*-pUC19 (Fig. 3*A*). We subsequently determined that Tris buffer slightly inhibits the metforminase reaction and switched to primarily using sodium phosphate, pH 8.0 (Fig. S2).

### mfmAB encodes a heteromeric metforminase

Since metforminase activity was detected in *E. coli* lysates expressing the *hypBAmfmAB* operon, we attempted to purify recombinant MfmA and MfmB proteins to use in enzyme assays. *mfmA* and *mfmB* were cloned into the pET28b plasmid to facilitate the expression of enzymes with *N*-terminal hexahistidine tags. Proteins were expressed in *E. coli* and enriched with nickel-affinity chromatography. We were able to produce highly enriched fractions of both enzymes, however, MfmA did not express as well and its yield and purity were less than those of MfmB (Fig. 3*B*). HPLC-based enzyme assays were used to assess enzyme activity. Reactions contained sodium phosphate, pH 8.0, 4 mM metformin, 2 mM NiCl_2_, and 5 μg of enzyme and were incubated at 37 °C for 2 h prior to analysis. Surprisingly, no metforminase activity was detected when MfmA and MfmB were combined in enzyme assays, or when each was assayed alone (Fig. 3*C*).

Since some heteromeric enzymes must be coexpressed to produce an active enzyme (44), we cloned *mfmAB* together into pET28b to facilitate the expression of MfmA with an *N*-terminal hexaistidine tag and native MfmB. Even though only the MfmA contained a polyhistidine tag, both proteins were highly enriched following nickel-affinity chromatography (Fig. 3*B*), indicating that MfmAB assumes a heteromeric quaternary structure that is very stable. The addition of 0.5 mM NiCl_2_ to cultures at induction resulted in enrichments that were slightly purer (Fig. 3*B*). Interestingly, the MfmB band was consistently more intense than that of MfmA, suggesting that MfmAB forms a heteromeric quaternary structure of uneven stoichiometry. Metforminase activity was detected when coexpressed MfmAB was assayed by HPLC as described above (Fig. 3*C*). The activity of MfmAB was slightly higher when nickel was added to cultures when inducing protein expression (Fig. 3*C*).

Enzyme assays were also analyzed by LC-MS to confirm guanylurea as a reaction product. The addition of MfmAB to an ammonium bicarbonate buffered solution containing metformin and nickel resulted in the appearance of a guanylurea [M + H] parent ion at *m*/*z* 103.1 that had the same retention time as an authentic guanylurea standard (Fig. 3*D*). Production of guanylurea by MfmAB was shown to be metal-dependent, as omitting nickel or adding 5 mM EDTA instead of metal resulted in the very little conversion of metformin to guanylurea (Fig. 3*E*).

We set up assays with various divalent cations to test whether metals other than nickel can stimulate metforminase activity. Reactions contained sodium phosphate, pH 8.0, 4 mM metformin, 2 mM metal, and 5 μg of MfmAB expressed without added nickel, and were incubated at 37 °C for 2 h prior to analysis. Maximum activity was observed with nickel, followed by cobalt and manganese (Fig. 3*F*). A small but detectable activity was observed with the addition of iron or magnesium while copper and molybdenum had no detectable activity (Fig. 3*F*). Since our feeding studies suggested that our strains have the ability to catabolize biguanide (Fig. 2), it was tested as a substrate for MfmAB using a spectrophotometric assay described below. We did not detect activity against biguanide in assays containing MfmAB.

### bguH encodes a novel biguanide aminohydrolase

**Figure 4:**
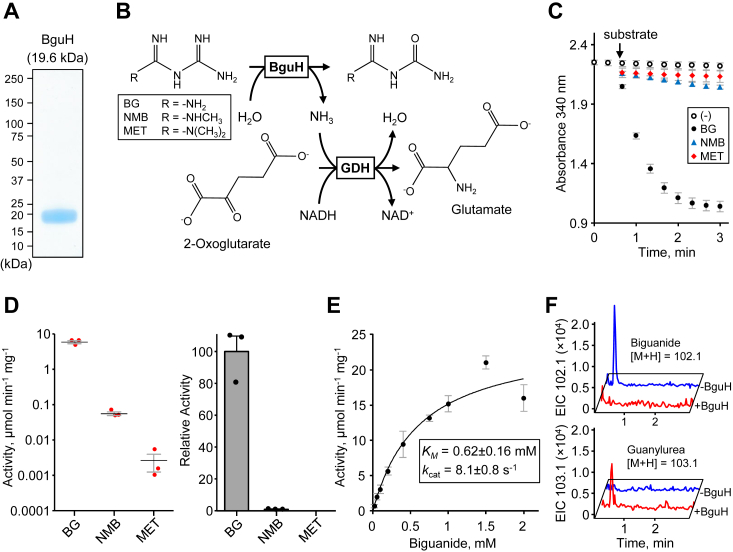
**Biguanidase activity of BguH enzyme.***A*, purity of enriched recombinant BguH enzyme expressed in *E. coli*. *B*, depiction of the spectrophotometric coupled assay used to measure BguH enzyme activity. BG, biguanide; GDH, glutamate dehydrogenase; MET, metformin; NMB, *N*-methylbiguanide. *C*, results of spectrophotometric assays (100 μl total volume, 25 °C) that contained 100 mM potassium phosphate, pH 8.0, 0.5 mM NADH, 1.5 U GDH, 0.5 mM two-oxoglutarate, either 5 μg (BG) or 50 μg (NMB, MET) BguH, and were started by adding 0.2 mM substrate or water (−). The arrow indicates substrate addition. Data represents mean and S.E.M. of 3 independent experiments. *D*, initial velocities and relative activities of BguH at 0.2 mM substrate. Assays were as described above. Data represents the mean and S.E.M. of three independent experiments. *E*, Michaelis–Menten kinetics of BguH against biguanide. Assays were as described above except 1.2 μg BguH was used and substrate concentrations were as indicated. Data represents the mean and S.E.M. of three independent experiments. *F*, LC-MS analysis of the BguH reaction. Assays (100 μl total volume, 25 °C) contained 100 mM ammonium bicarbonate, pH 8.0, 2 mM biguanide, without (*blue* traces) or with 1 μg BguH (*red* traces), and were incubated for 1 h prior to LC-MS analysis. Extracted ion chromatograms (EIC) shown represent the biguanide [M + H] = 102.1 and guanylurea [M + H] = 103.1 molecular ions.

Our *Pseudomonas* isolates readily grew on biguanide as a nitrogen source, suggesting enzyme-mediated breakdown. Since MfmAB had no detectable activity against biguanide in our assays, we decided to test BguH for activity. The coding sequence was cloned into the pET28b plasmid to facilitate expression of *N*-terminally hexahistidine tagged enzyme. Protein was expressed in *E. coli* and enriched with nickel-affinity chromatography, which resulted in nearly homologous BguH preparations (Fig. 4*A*). We optimized a reporter assay to detect biguanide hydrolase activity. The reporter assay uses commercially available glutamate dehydrogenase (GDH) and is based on the stoichiometric reduction of NAD^+^ to NADH, which can be measured by its distinctive absorbance at 340 nm (Fig. 4*B*). The addition of 0.2 mM biguanide to a reaction mixture containing 5 μg BguH caused the absorbance at 340 nm to rapidly decrease compared to the control (Fig. 4*C*), indicating that BguH has aminohydrolase activity against biguanide. The addition of metformin or *N*-methylbiguanide to 0.2 mM caused the absorbance at 340 nm to slowly decrease compared to the control, but BguH was increased to 50 μg in these assays (Fig. 4*C*). We measured a specific activity at 0.2 mM biguanide of 5.9  ± 0.6 μg min^-1^ mg^-1^ (Fig. 4*D*). The activity against 0.2 mM of either *N*-methylbiguanide or metformin was 0.056  ± 0.007 μg min^-1^ mg^-1^ and 0.003  ± 0.001 μg min^-1^ mg^-1^, which corresponds to ∼1% or ∼0.05% of the activity against biguanide, respectively (Fig. 4*D*). Since BguH strongly prefers biguanide over structurally related substrates, we further optimized the reporter assay and determined that BguH has a *K*_*M*_ of 620  ± 160 μM and a *k*_cat_ of 8.1  ± 0.8 s^−1^ against biguanide (Fig. 4*E*).

The reporter assay described above showed that BguH releases ammonia from biguanide, but didn’t confirm that the other reaction product is guanylurea, as expected. To confirm the production of guanylurea, enzyme assays were analyzed by LC-MS with authentic standards of biguanide and guanylurea. We were unable to effectively separate biguanide and guanylurea chromatographically with our LC-MS as configured. Since our analysis was performed on a single quadrupole mass spectrometer with a nominal mass resolution, and the difference in guanylurea and biguanide [M + H] parent ions is 1.0, there was some overlap between the substrate and product peaks due to natural isotope abundance. Still, we were able to detect a significant loss of the biguanide [M + H] peak at m/z 102.1 and the appearance of the expected guanylurea [M + H] peak at m/z 103.1 that was dependent on BguH addition (Fig. 4*F*). Together, the reporter assay and LC-MS analysis indicate that BguH has aminohydrolase activity against biguanide and the reaction products are guanylurea and ammonia.

### Overexpression of bguH allows *E. coli* to use biguanide as a nitrogen source

**Figure 5:**
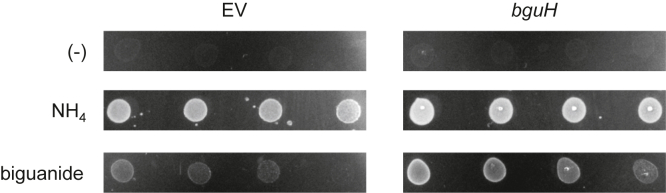
**Expression of *bguH* enables *E. coli* to grow on biguanide.***E. coli* MG1655 harboring pUC19-*bguH* or empty vector (EV) were grown overnight in M9 minimal medium, washed thrice with minimal medium lacking nitrogen, and diluted to an optical density of 1.0, 0.2, 0.04, and 0.08. 5 μl of each dilution was spotted on minimal medium plates containing no nitrogen (−) or with 20 mM ammonium chloride or biguanide and grown at 37 °C for 16h prior to imaging.

To confirm that *bguH* encodes a biguanide aminohydrolase, we tested the effect of overexpression on the ability of *E. coli* to use biguanide as the sole nitrogen source. *E. coli* MG1655 harboring *bguH*-pUC19 or empty vector was plated on M9 minimal medium lacking nitrogen. Neither strain grew on the medium without added nitrogen, confirming that our base medium lacked sufficient nitrogen to sustain growth (Fig. 5). When ammonium chloride was added to reconstitute standard M9 medium, both strains grew similarly (Fig. 5). *E. coli* harboring an empty vector showed very little growth on medium supplemented with biguanide, while *E. coli* overexpressing *bguH* grew readily (Fig. 5). These results show that overexpressing *bguH* allows *E. coli* to grow on biguanide and confirms a role for *bguH* in biguanide breakdown.

## Discussion

This study provides significant insights into the metabolic capacities of environmental Pseudomonads to degrade metformin. We validated that *mfmAB* encodes a heteromeric metforminase, which completes a pathway for complete metformin assimilation. Three recently published studies also showed that *mfmAB* homologs from other environmental isolates encode metforminases and they all show very similar biochemical characteristics as ours (37, 38, 39). Coexpression of *mfmAB* was necessary for the production of active enzyme; mixing MfmA and MfmB expressed individually did not reconstitute enzyme activity (Fig. 3*C*). The dependence on coexpression is now observed by four independent research groups and suggests that the presence of both peptides is necessary for proper protein folding or assembly into the proper quaternary structure. The MfmAB complex must be tightly bound because native MfmB was enriched by nickel-affinity chromatography even though only MfmA was polyhistidine-tagged (Fig. 3*B*). Other heteromeric enzymes require coexpression to form an active complex and can be co-purified by tagging one subunit, such as the prokaryotic five-oxoprolinase PxpBC (44) and bacterial luciferase LuxAB (45, 46). SDS-PAGE analysis of enriched MfmAB showed that MfmB was more abundant than MfmA (Fig. 3*B*), suggesting that the active metforminase may be a heteromeric complex of uneven stoichiometry. This is consistent with recently published crystal structures of MfmAB, which show that two MfmB subunits and one MamA subunit form an uneven heterotrimer (37, 38, 39). One study concluded that the heterotrimer, MfmAB_2_ is a functional metforminase (37). Two other studies determined that active metforminase is a heterohexamer consisting of a dimer of trimers, 2(MfmAB_2_) (38, 39). An uneven heterotrimer appears to be the basic functional unit with larger quaternary aggregates also forming.

Recombinant MfmAB had robust metforminase activity and the enzyme is highly specific for metformin. We did not detect activity against biguanide, and other recent studies have shown that MfmAB strongly prefers metformin over structurally related analogs (37, 38, 39). We show that MfmAB converts metformin to guanylurea (Fig. 3, *C*–*F*). The other expected reaction product is dimethylamine, and although we didn’t explicitly measure dimethylamine, other recent studies confirm it as a reaction product (37, 38, 39). We expected metal dependence based on the fact that MfmA and MfmB are both ureohydrolase protein family members that typically catalyze metal-dependent hydrolysis reactions (47, 48). *mfmAB* is consistently found in an operon with genes encoding predicted nickel incorporation proteins, *hypAB*, suggesting nickel dependence of MfmAB. We confirmed the metal dependence of MfmAB (Fig. 3*E*) and observed maximum enzyme activity with nickel (Fig. 3*F*). Cobalt and manganese were the only other divalent cations that stimulated activity to at least 5% that of nickel (Fig. 3*F*). Ureohydrolase protein family members such as agmatinase and arginase typically catalyze hydrolysis reactions of guanidinium moieties resulting in urea production (41, 49, 50). The release of dimethylamine instead of urea is unique for MfmAB, which appears to have recently evolved from close homologs with dimethylguanidine hydrolase activity (39).

The recent evolution of *mfmAB* is a remarkable ecological occurrence. A few years ago, metformin degradation could only be achieved through community effort (51). Bacteria were isolated that could degrade guanylurea but not metformin until recently when bacteria capable of complete metformin assimilation were isolated from four independent groups across three continents (33, 37, 38, 39). All of these isolates encode *mfmAB* in a conserved metforminase operon (Fig. 1). Metforminase likely evolved in *Aminobacter*, where it is encoded in the bacterial chromosome, and incorporation of the genes into bacterial plasmids allowed its rapid spread to other *Aminobacter* and *Pseudomonas* species already possessing the capacity to degrade guanylurea. The conservation of a metforminase operon in all reported metformin-utilizing bacterial isolates suggests that *mfmAB* is now the primary route of environmental metformin breakdown worldwide.

We also identified a novel biguanide hydrolase gene *bguH* on the same ∼80-kb plasmids as *mfmAB*, although not in the same metforminase operon (Fig. 1). We show that recombinant BguH has robust activity against biguanide (Fig. 4*E*) and that ammonia (Fig. 4, *C*–*E*) and guanylurea (Fig. 4*F*) are the reaction products. We detected minor aminohydrolase activity against *N*-methylbiguanide and metformin, but these were less than 1% of the activity against biguanide (Fig. 4*D*). Expression of *bguH* in *E. coli* allowed biguanide to be used as a nitrogen source (Fig. 5), further confirming the biguanide hydrolase activity of BguH. Growth is likely due solely to ammonia release, as *E. coli* are not expected to metabolize guanylurea. Nucleoside deaminases are the closest homologs of BguH (52), but its evolutionary history is unclear as there are no sequences in the NCBI database that have greater than 60% amino acid identity. The *bguH* gene occurs in our isolates and in another Pseudomonad isolated from Minnesota, but not in recently identified metformin-degrading bacteria isolated from Germany or China, suggesting it evolved recently and not in connection to *mfmAB*.

Biguanide is not an intermediate of the primary MfmAb-dependent pathway of metformin breakdown (Fig. 1). However, it could arise from successive *N*-demethylations of metformin. Prior to the recent evolution and spread of *mfmAB*, metformin demethylation to biguanide may have been a significant means of breakdown in microbial communities, and microbes that use *N*-methylbiguanide as a nitrogen source in addition to our isolates have been reported (34). *N*-methylbiguanide use could be viewed as evidence for an alternate metformin breakdown pathway. However, we show that BguH can deaminate *N*-methylbiguanide, albeit at ∼1% the rate of biguanide deamination (Fig. 4, *C* and *D*), and this could account for the slow growth observed on *N*-methylbiguanide (Fig. S1). Caffeine is similarly broken down by a pathway involving successive *N*-demethylations (53, 54). There are other parallels between caffeine and metformin breakdown. These compounds are the top two anthropogenic pollutants in waterways worldwide (23) and different environmental Pseudomonads have evolved that are capable of their breakdown (37, 38, 53). In the case of caffeine degraders, *ndmABC* genes encode caffeine *N*-demethylases (53, 54). The KHPS2 isolate has homologs of *ndmABC* genes in its chromosomal genome, but no homologs occur in KHPS1, so we did not consider them a good candidate metformin *N*-demethylases. Still, metformin *N*-demethylation, along with this newly discovered biguanide hydrolase activity, would complete an alternate metformin breakdown pathway. Biguanide could also come from the partial breakdown of other biguanide derivatives such as the metformin predecessors phenformin and buformin (55, 56), antimalarial drugs such as Proguanil (57), and disinfectants such as Chlorhexidine and Polyaminopropyl biguanide (58, 59). Having a complete *mfmAB*-initiated metformin breakdown pathway and *bguH* may allow microorganisms to utilize a variety of biguanide-related compounds.

We initially searched for metformin-degrading microorganisms in primary sludge from the Saint Paul Water Treatment Plant because we predicted that metforminase may have evolved in the human gut or in sewage systems where metformin levels are consistently high. The isolation of other metformin degraders from wastewater treatment plants around the world seems to support this notion (33, 34, 35, 36). Regardless, the existence of a metforminase on mobile elements found around the world suggests that metforminase activity could begin to play a role in human gut microbiota. Identification of a complete metformin/biguanide breakdown pathway will allow researchers to test whether these genes occur in gut microbiota and if their presence changes the effectiveness of metformin as a blood sugar-lowering agent.

## Experimental procedures

### Chemicals and reagents

Metformin-HCl was from AstaTech. Biguanide-HCl was from AmBeed. L-Glutamic dehydrogenase from bovine liver was from Sigma Aldrich. All other chemicals were from Sigma Aldrich.

### Bioinfomatic analyses

Genomic analyses of *Pseudomonas* sp. KHPS1 (GenBank Accessions: CP100551.1, CP100552.1) and *P. hydrolytica* strain KHPS2 (GenBank Accessions: CP100553.1, CP100554.1) were performed with Galaxy software and its tools (60). Additional sequences were obtained from the National Center for Biotechnology Information databases (61).

### *Pseudomonas* nitrogen source growth assays

Single colonies from fresh plates of *Pseudomonas* sp. KHPS1 and *P. hydrolytica* strain KHPS2 were grown overnight in LB medium and washed three times with citrate-acetate minimal medium lacking nitrogen (30). Cells were diluted to an optical density of 1.0, 0.2, and 0.04 and 5 μl of each dilution was spotted on minimal medium plates lacking nitrogen or containing 1 mM of metformin, guanylurea, guanidine, or biguanide as the only nitrogen source. Plates were incubated for 24 h at 37 °C prior to imaging.

### Gene cloning

The *hypBAmfmAB* operon was cloned into pUC19 using Gibson Assembly (New England Biolabs). pUC19 was PCR amplified using oligonucleotide primers 1 and 2 (All oligonucleotide primers are listed in Table S1) and *hypBAmfmAB* was amplified with primers 3 and 4. Amplicons were used for Gibson Assembly according to the manufacturer’s recommendations. The BguH coding sequence was PCR amplified with primers 5 and 6, treated with restriction enzymes HindIII and XbaI, and ligated into the corresponding sites of pUC19.

For protein expression, the *Pseudomonas* sp. KHPS1 genes *mfmA* (primers 7 and 8), *mfmB* (primers 9 and 10), and mfmAB (primers 11 and 12) were PCR amplified, amplicons were treated with restriction enzymes NheI and XhoI (*mfmA*), NdeI and EcoRI (mfmB), or NheI and EcoRI (mfmAB), and ligated into the corresponding sites of pET28b. The BguH coding sequence was optimized for *E. coli* expression (Fig. S3) and synthesized by Integrated DNA Technologies gBlocks service. The fragment was PCR amplified with primers 13 and 14 and the amplicon was treated with NdeI and HindIII and ligated into the matching sites of pET28b.

### Protein expression and purification

Optimization of protein expression was performed as previously described (62, 63). For the production of recombinant MfmA, MfmB, MfmAB, and BguH, the corresponding expression vector was transformed into *E. coli* strain BL21-(DE3)-RIPL. Positive transformants were grown at 37 °C in 200 ml LB medium containing 50 μg/ml kanamycin. Cultures were cooled to 20 °C when the absorbance at 600 nm reached 0.8, and isopropyl β-d-thiogalactoside (ITPG) and ethanol were added to final concentrations of 0.5 mM and 4% (v/v), respectively. As indicated, some MfmAB-expressing cultures also had 0.5 mM NiCl2 added along with IPTG and ethanol. Incubation was then continued overnight at 22 °C. Cell lysates were prepared by harvesting cells by centrifugation (5000*g*, 10 min, 4 °C) and suspending pellets in 6 ml of lysis buffer (50 mM Tris-HCl, pH 8.0, 300 mM NaCl, 10 mM imidazole). Cells were sonicated using a Braun-Sonic 2000 set to 50% power for six, 15 s pulses, cooling on ice for 60 s between pulses. The resulting lysate was centrifuged at 28,000*g* for 10 min. The supernatant was added to a column containing 0.30 ml of HisPur Ni-NTA resin (Thermo Fisher Scientific) and washed with 9 ml of wash buffer (50 mM Tris-HCl, pH 8.0, 300 mM NaCl, 20 mM imidazole). Recombinant proteins were eluted with 0.50 ml of elution buffer (50 mM Tris-HCl, pH 8.0, 300 mM NaCl, 200 mM imidazole). Amicon Ultra-4 10k MWCO spin columns were used to concentrate proteins and exchange buffer with 100 mM KCl, 50 mM Tris-HCl, pH 8.0. Glycerol was added to a final concentration of 10% (v/v), and 10 μl aliquots were snap-frozen in liquid nitrogen and stored at −80 °C. SDS-PAGE was used to assess the purity of our enrichments.

### Enzyme assays

Assays of whole cell lysates (Fig. 3*A*). *E. coli* strain BL21-(DE3)-RIPL harboring pUC19-*hypBAmfmAB*, pUC19-*bguH*, or empty vector, was grown at 37 °C in 200 ml LB medium containing 50 μg mL^−1^ kanamycin. When the absorbance at 600 nm reached 0.8, cultures were cooled to 20 °C, and IPTG, ethanol, and NiCl_2_ were added to final concentrations of 0.5 mM, 4% (v/v), and 500 μM, respectively. Incubation was then continued overnight at 22 °C. Cell lysates were prepared by harvesting cells by centrifugation (8000*g*, 10 min) and suspending pellets in 7 ml of lysis buffer (50 mM Tris-HCl, pH 8.0, and 300 mM NaCl). Cells were sonicated using a Braun-Sonic 2000 set to 50% power for eight, 15 s pulses, cooling on ice for 60 s between pulses. The resulting lysates were centrifuged at 20,000*g* for 10 min and concentrated using Amicon Ultra-4 10k MWCO spin columns to a final volume of 200 μl. Samples were diluted to 1.9 μg μL^−1^ with lysis buffer. Assays (100 μl) contained 50 mM Tris-HCl, pH 8.0, 4 mM metformin, 0.5 mM NiSO_4_, 50 μM FeCl_3_, 5 μM MnCl_2_, 12.4 μM ZnCl_2_, 2.52 μM CuCl_2_, 2.5 μM CoCl_2_, 2.5 μM Na_2_MoO_4_, 2 mM MgCl_2_, 50 μM CaCl_2_, and were started by adding 50 μl (95 μg total protein) freshly-prepared lysate. Reactions were incubated at 37 °C for 1.5 h 10 μl of reaction was analyzed with an Agilent 1100 series HPLC with a G1315B DAD detector using a Hypersil GOLD 250 × 4.6 mm C18 column (ThermoFisher Scientific) with 25% 10 mM potassium phosphate, pH 6.5; 75% acetonitrile as the mobile phase at a flow rate of 1 ml min^−1^. Compounds were detected at 220 nm (guanylurea) and 234 nm (metformin). The amount of compound in each peak was determined by integrating peak areas using OpenLab ChemStation software version 2.19.20, and comparing values to those obtained from standard curves prepared for guanylurea and metformin.

HPLC-based metforminase assays (Fig. 3, *C* and *F*). Assays (100 μl) contained 125 mM sodium phosphate, pH 8.0, 4 mM metformin, 2 mM NiCl_2_, and were started by adding 5 ug of enzyme. Reactions were incubated at 37 °C for 2 h and analyzed by HPLC as described above.

LC-MS-based metforminase assays (Fig. 3, *D* and *E*). Assays (100 μl) contained 100 mM NH_4_HCO_3_, pH 8.0, 4 mM metformin, without or with 2 mM NiCl_2_, 5 mM EDTA, and 5 μg MfmAB. Reactions were incubated at 37 °C for 2 h and stopped by adding 2 μl 1M HCl and the mixture was diluted 1:20 in water. LC-MS analysis was performed on an Agilent 1260 Infinity II and single quadruple MSD with a 2.1 × 50 mm reverse-phase SB-C18 column (1.8 μm), with 0.1% formic acid as the mobile phase at a flow rate of 0.4 ml min^−1^. Samples were ionized *via* electrospray, and MS was run on positive mode with a capillary voltage of 3000 V. [M + H] abundances for each compound (metformin: 130.1 *m/z*, guanylurea: 103.1 *m/z*) were integrated over the course of the reaction and compared against standard curves to determine concentrations.

Spectrophotometric biguanidase assays (Fig. 4, *C* and *D*). Assays (100 μl) contained 100 mM potassium phosphate, pH 8.0, 0.5 mM NADH, 0.5 mM 2-oxoglutarate, 1.5 U glutamate dehydrogenase, and either 5 μg (biguanide) or 50 μg (*N*-methylbiguanide and metformin) BguH. The mixture was transferred to 100 μl quartz cuvettes and reactions were started by adding water or 0.2 mM biguanide, *N*-methylbiguanide, or metformin and mixing rapidly while recording the absorbance at 340 nm. For determining the kinetic parameters of BguH, assays were performed in a similar manner except that assays contained 1.2 μg BguH and were started by adding various biguanide concentrations. Kinetic parameters were calculated by fitting data to the Michaelis–Menten equation using GraphPad Prism software version 5.01.

LC-MS-based biguanidase assays (Fig. 4*F*). Assays (100 μl) contained 100 mM NH_4_HCO_3_, pH 8.0, 2 mM biguanide, without or with 1 μg BguH. Reactions were incubated at 37 °C for 1 h and stopped by adding 5 μl 1M HCl. The mixture was filtered with a 3000 MWCO microcentrifuge filter unit to remove protein and the filtrate was diluted 1:20 in water. LC-MS analysis was performed on an Agilent 1260 Infinity II and single quadruple MSD with a 2.1 × 50 mm reverse-phase SB-C18 column (1.8 μm), with 0.1% formic acid as the mobile phase at a flow rate of 0.4 ml min^−1^. Samples were ionized *via* electrospray, and MS was run on positive mode with a capillary voltage of 3000 V. [M + H] abundances for each compound (biguanide: 102.1 *m/z*, guanylurea: 103.1 *m/z*) were integrated throughout the reaction and compared against standard curves to determine concentrations.

### Growth assays with *E. coli*

*E. coli* MG1655 harboring pUC19-*bguH* or empty vector were grown overnight at 37 °C in M9 minimal medium with 0.4% (w/v) glucose. Cells were washed twice with M9 media that did not contain any nitrogen and then diluted to OD_600_ of 1.0. This was then serially diluted to OD_600_ of 0.2, 0.04, and 0.008. 5 μl of each dilution was spotted onto M9 minimal medium plates with 0.4% (w/v) glucose, 0.5 mM IPTG, and either no nitrogen or 20 mM of either biguanide or ammonium chloride. Plates were then incubated for 16 h at 37 °C prior to imaging.

## Data availability

All data is either contained within the manuscript or will be shared upon request by contacting Thomas Niehaus (tniehaus@umn.edu).

## Supporting information

This article contains supporting information.

## Conflict of interest

The authors declare that they have no conflicts of interest with the contents of this article.
