# Supplementary material for: Biochemical and genomic evidence for converging metabolic routes of metformin and biguanide breakdown in environmental Pseudomonads
Source: J Biol Chem. 2024 Oct 28;300(12):107935. doi: 10.1016/j.jbc.2024.107935 (PMC11647477; doi:10.1016/j.jbc.2024.107935)
Supplement: Supporting information [file mmc1.pdf]

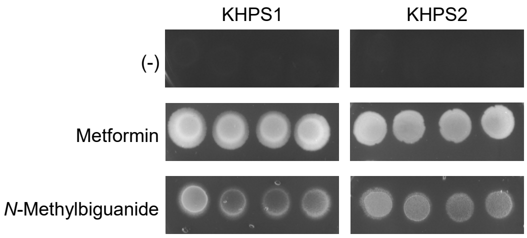

Supplemental Figure 1. Our isolates use *N*-methylbiguanide as a nitrogen source.

Pseudomonas sp. KHPS1 and *Pseudomonas hydrolytica* strain KHPS2 were grown overnight in LB medium and washed thrice with citrate-acetate minimal medium lacking nitrogen. Each overnight was diluted to an optical density of 1.0, 0.2, 0.04, and 0.008 and 5 µL was spotted on minimal medium plates lacking nitrogen (-) or plates containing 1 mM of metformin or 1 mM *N*-methylbiguanide as the sole nitrogen source. Plates were incubated for 48 hours at 37°C prior to imaging.

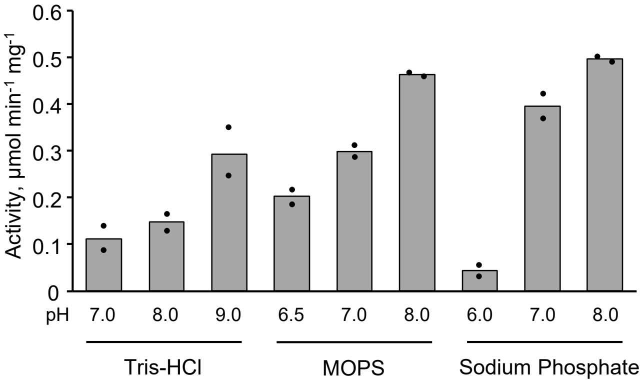

Supplemental Figure 2. Effect of buffer and pH on MfmAB enzyme activity.

Assays (100 µL) contained either 50 mM Tris-HCl, 50 mM MOPS, or 125 mM sodium phosphate at the indicated pH, 4mM Metformin, 2mM $NiCl_2$, and 5 µg MfmAB. Reactions incubated for 2 hours at 37°C and analyzed by HPLC as described in the methods. Data represents the mean of two independent experiments.

>BguH CDS (codon optimized for *E. coli*)

ATGTCAGAACGTAACCATGAGAAGGGAATGTTGCTGGCTATTGAACAGGCGCTTAAAGCTG
TCGCTTCTGGGGAGCCACCATTTGGCGTGGTTATTCTGGACGCGCAGGGTGAGTTAGTTG
CTGCTACTCATGACGAAGTGAACACCCGCGGTGATATGTCGGCCCACGCTGAAACCCTTG
CTGTGCGTGCAGCGTGCCAAGTGCGCGGGCCATCGTTAGAGGGGTGTTTTTTGTACACGA
CGTGCGAACCATGTCCCATGTGTTTTACCACTGCTTGGTTGGCACGTATCGGGGGCGTCG
TTTACGCTACGACTATGGATGAGGTTCATAAGATTTTGGGGGATGCCCAACGCGAACTGCG
TGTGCCCGTGACCCAAATGAATGATCTGTCCGGCGAGCCTGTGACTTTAGTAAAAGGAGTA
TTGCGCGACCGCTGCTTACAATTATTTCGTGAGCACGCTGCTACCCTGGCTACCACAAAAT
GA

Supplemental Figure 3. The BguH coding sequence used in this study.

Supplemental Table 1. Oligonucleotide Primers Used in This Study

complementation constructs		
primer	name	sequence (5'->3')
1	Met puc19 P1	gcacatcgcttcggccatacctcgaattcactggccgtcg
2	Met puc19/pGEM P2	gcatccttgccgaaatgcagggatcctctagagtcgacctg
3	Met plasmid operon F	ctgcatttcggcaaggatgc
4	Met plasmid operon R	gtatggccgaagcgatgtgc
5	BguH_HindIII_F	aaggaagcttatgtcagaacgtaaccatgagaaggg
6	BguH_XbaI_R	aaggtctagatcattttgtggtagccagggtagc
expression constructs		
7	gbuA Nhe F	ggaattcgctagcatgcttgatcgaaaaacagagacagc
8	gbuA Xho s R	ttccgctcgagttacggttgcttgccctgctg
9	gbuB Nde F	ggaattccatatgaacccagcaaaatcctatgcc
10	gbuB Eco R	cggaattcctattcggcggcgaccagatc
11	GbuA Nhe F	ggaattcgctagcatgcttgatcgaaaaacagagacagc
12	GbuB Eco R	cggaattcctattcggcggcgaccagatc
13	BguH Nde F	ggaattccatatgtcagaacgtaaccatgagaag
14	BguH Hind R	cccaagctttcattttgtggtagccagggtag
